# Case Report: Multimodal imaging diagnosis of arrhythmogenic cardiomyopathy complicated with thrombosis

**DOI:** 10.3389/fcvm.2026.1981891

**Published:** 2026-10-29

**Authors:** Ziming Liu, Xiaoyang Liu, Xiuyun Luo, Wei Miao

**Affiliations:** 1 School of Medical Imaging, Shandong Second Medical University, Weifang, China; 2 Department of Ultrasonography, Weifang People's Hospital, Weifang, China

**Keywords:** arrhythmogenic cardiomyopathy, case report, echocardiography, multimodal imaging, right ventricular thrombus

## Abstract

**Background:**

Arrhythmogenic Cardiomyopathy (ACM) is an inherited cardiomyopathy, and concomitant intracardiac thrombosis is a relatively rare clinical complication, whose pathogenesis is linked to blood flow stasis, endothelial injury and impaired cardiac function. Timely and accurate diagnosis and standardized treatment of this complication remain critical to avoid embolic adverse events.

**Case summary:**

We present a case of a 37-year-old Asian female patient with ACM complicated with multiple thrombi at the right ventricular apex. She was hospitalized with a 3-month history of recurrent chest tightness and dyspnea aggravated in the latest half month. The electrocardiogram showed sinus rhythm with complete right bundle branch block, marked right axis deviation, ST-segment depression and T-wave inversion in leads V1–V3, premature ventricular complexes, and prolonged QT interval, which may suggest elevated right ventricular pressure load; laboratory examinations suggested cardiac dysfunction. Transthoracic echocardiography detected right ventricular dilatation, diffuse apical trabeculation and mural thrombus. Cardiac magnetic resonance further verified abnormal right ventricular wall motion, non-enhanced intracardiac thrombus and late gadolinium enhancement supporting the diagnosis of ACM.

**Discussion:**

Transthoracic echocardiography enables early identification of cardiac structural lesions and thrombi, whereas cardiac magnetic resonance provides comprehensive evaluation of myocardial pathological changes. Combined application of the two imaging modalities is indispensable for confirming ACM with intracardiac thrombus. Early standardized anticoagulation combined with holistic therapy effectively lowers embolism risk and optimizes long-term prognosis. Clinicians ought to reinforce regular screening and targeted intervention for this rare thrombotic complication in ACM patients.

**Take home messages:**

Transthoracic echocardiography serves as a convenient first-line screening tool for detecting right-ventricular morphological abnormalities and intracardiac thrombus. The mechanism of thrombus formation in biventricular-involved ACM with predominant right-ventricular lesions may be speculated with reference to Virchow's triad. Post-discharge imaging follow-up verified thrombus resolution after timely anticoagulation.

## Introduction

Arrhythmogenic Cardiomyopathy (ACM) is a rare inherited cardiomyopathy characterized pathologically by progressive replacement of the myocardium with fibrofatty tissue. This leads to local or global abnormalities, mainly caused by mutations in the cardiac ryanodine receptor RyR2, desmoplakin, and the regulatory sequences of the transforming growth factor *β* gene (1). According to the 2020 Padua Criteria, ACM is currently categorized into right ventricular-predominant, biventricular, and left ventricular-predominant subtypes, with substantial heterogeneity in clinical phenotypes. In the early stage, there may be no obvious symptoms. Some patients present with palpitations and syncope as the initial manifestations. In the advanced stage, it can progress to right heart failure or even biventricular failure (2). However, the clinical situation of ACM combined with thrombosis is relatively rare. The reported incidence in existing literature is less than 5%. Complications such as pulmonary embolism and systemic embolism caused by thrombus detachment can significantly increase the clinical risk (3).

## Case presentation

A 37-year-old Asian female patient was admitted to the hospital on September 10, 2025 due to “repeated chest tightness and shortness of breath for more than 3 months, aggravated over the past half month”. The patient experienced chest tightness and shortness of breath without any obvious cause three months ago. The symptoms worsened with physical activity and did not improve after rest. She also suffered from intermittent nocturnal breathing difficulties and difficulty falling asleep. She came to the hospital for further treatment. After admission, the physical examination of the patient showed that the apex beat was displaced leftward and downward. No pathological murmurs were heard in any valve area, and no other obvious abnormalities were found. The patient had no obvious previous episodes of related symptoms before this admission. No significant positive social-history information was available from clinical interview. With respect to family-history investigation, both the patient's parents and other available family members had no history of arrhythmogenic cardiomyopathy, unexplained arrhythmia or sudden death, suggesting a sporadic origin of this case without definite hereditary predisposition. Laboratory tests indicated a hemoglobin level of 105 g/L, suggesting mild anemia, a uric acid level of 432 μmol/L, indicating hyperuricemia, a D-dimer level of 0.35 mg/L, and a NT-proBNP level of 680 pg/mL, suggesting cardiac dysfunction. The electrocardiogram demonstrated sinus rhythm with complete right bundle branch block (RBBB), characterized by a QRS duration ≥ 120 ms and rSR' pattern in leads V1–V2. There was marked right axis deviation. Notable ST-segment depression and T-wave inversion were observed in leads V1–V3, consistent with a right ventricular strain pattern often seen in the setting of RBBB and right heart overload. Additionally, premature ventricular complexes were present, and the QT interval appeared prolonged. These findings, particularly the combination of RBBB, right axis deviation, and anterior T-wave inversions, may suggest right ventricular pressure overload (Figure 1). 24-hour Holter monitoring showed no evidence of non-sustained ventricular tachycardia (NSVT) or sustained ventricular tachycardia (VT). The patient had no prior history of syncope. During exercise testing, the patient's breath-holding symptom slightly worsened, the frequency of premature ventricular complexes (PVCs) increased and paired PVCs were observed, though non-sustained VT was not provoked; these findings may indicate an elevated arrhythmic risk. Considering the patient's clinical symptoms and electrocardiographic features, the possibility of hemodynamic instability triggered by ventricular arrhythmias should be considered. An implantable cardioverter-defibrillator (ICD) may reduce the risk of sudden cardiac death. However, from a long-term prognostic perspective, progressive right-ventricular fibrosis may lead to gradual reduction of R-wave amplitude and compromise ICD sensing stability. Based on the patient's clinical manifestations and auxiliary examinations, the patient has no obvious risk factors and is not a pathogenic gene carrier, with a low probability of malignant arrhythmia. ICD implantation is not indicated at present, and regular follow-up surveillance is recommended. In addition, potential long-term risks including age-related concerns, infection and device malfunction should also be weighed.

**Figure 1:**
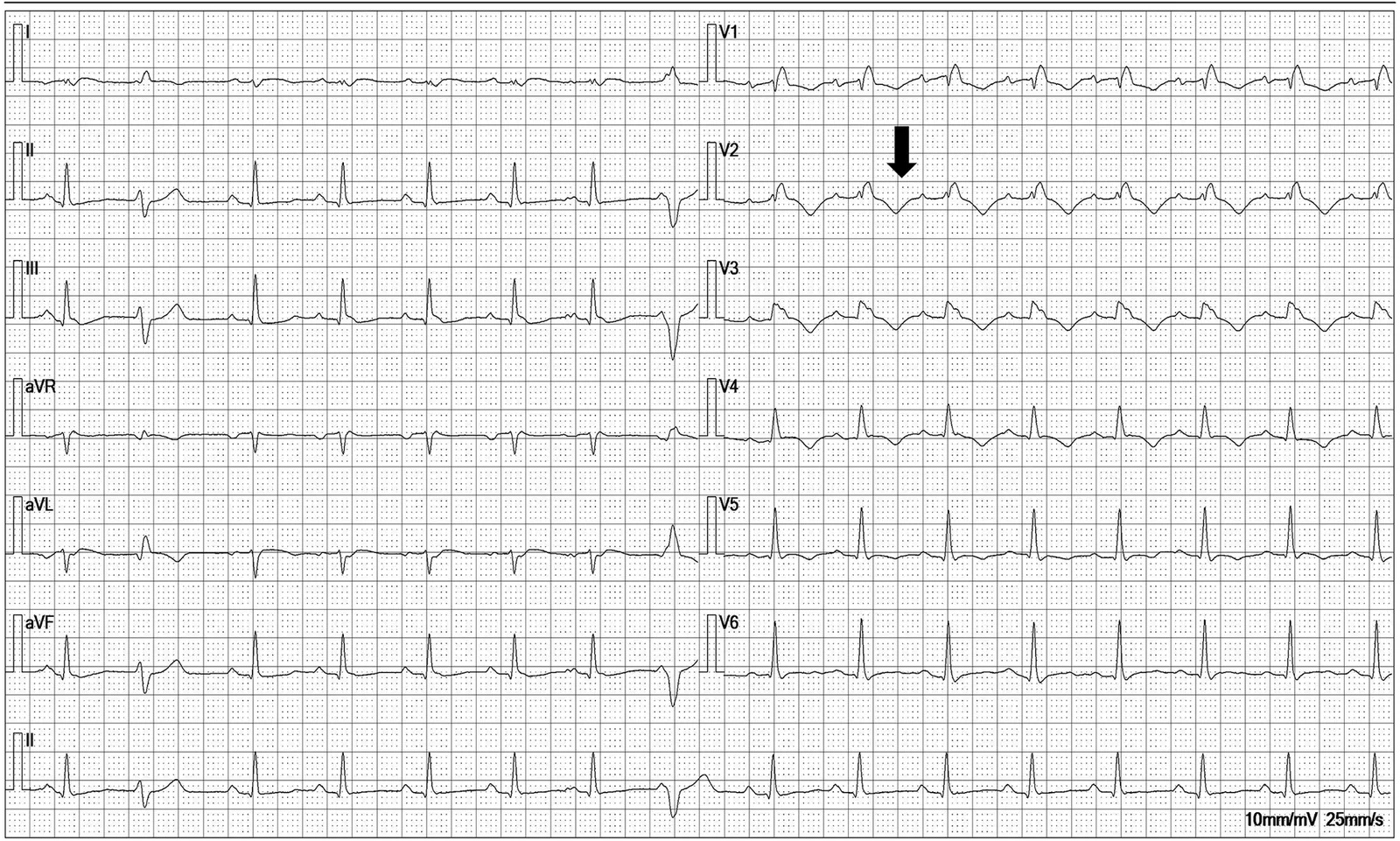
The 12-lead surface electrocardiogram shows sinus rhythm with complete right bundle-branch block (RBBB), featuring QRS duration ≥120 ms and an rSR' pattern in leads V1-V2. Marked right-axis deviation is present. T-wave inversion (black arrows), ST-segment depression, and QT-interval prolongation are noted in leads V1-V3, consistent with a right-ventricular strain pattern related to RBBB and right-heart overload. Ventricular premature complexes can also be observed.

Chest computed tomography (CT) demonstrated cardiomegaly and pericardial thickening, with a diagnosis of cardiomegaly and small pericardial effusion. Transthoracic echocardiography using multiple acoustic windows demonstrated that the left ventricular end-diastolic anteroposterior diameter was 40.1 mm, the left ventricular ejection fraction (LVEF) was approximately 40%, the left ventricular fractional shortening (FS) was about 23%, the right ventricular transverse diameter was approximately 33.6 mm, the right atrial transverse diameter was about 56.0 mm, the lateral tricuspid annular systolic velocity (TDI S’) was approximately 7 cm/s, the right ventricular fractional area change (FAC) was approximately 33%, and the tricuspid annular plane systolic excursion (TAPSE) was about 16 mm. Diffuse myocardial trabeculation could be observed at the right ventricular apex. Within these formations, two hyperechoic masses could be seen, with dimensions of approximately 14.4 mm × 12.2 mm and 9.6 mm × 9.9 mm respectively. The boundaries were clear, attached to the ventricular wall, and did not show significant movement with heartbeats. The tricuspid valve had poor closure and moderate eccentric regurgitation signals were detected. Its peak flow rate: 2.36 m/s, peak gradient (PG): 22 mmHg. Echocardiography revealed structural and motion abnormalities of the right ventricular apex with multiple apical mural thrombi (Figures 2A,B). Combined with clinical findings, ACM could not be excluded, and further cardiac magnetic resonance (CMR) evaluation was recommended.

**Figure 2:**
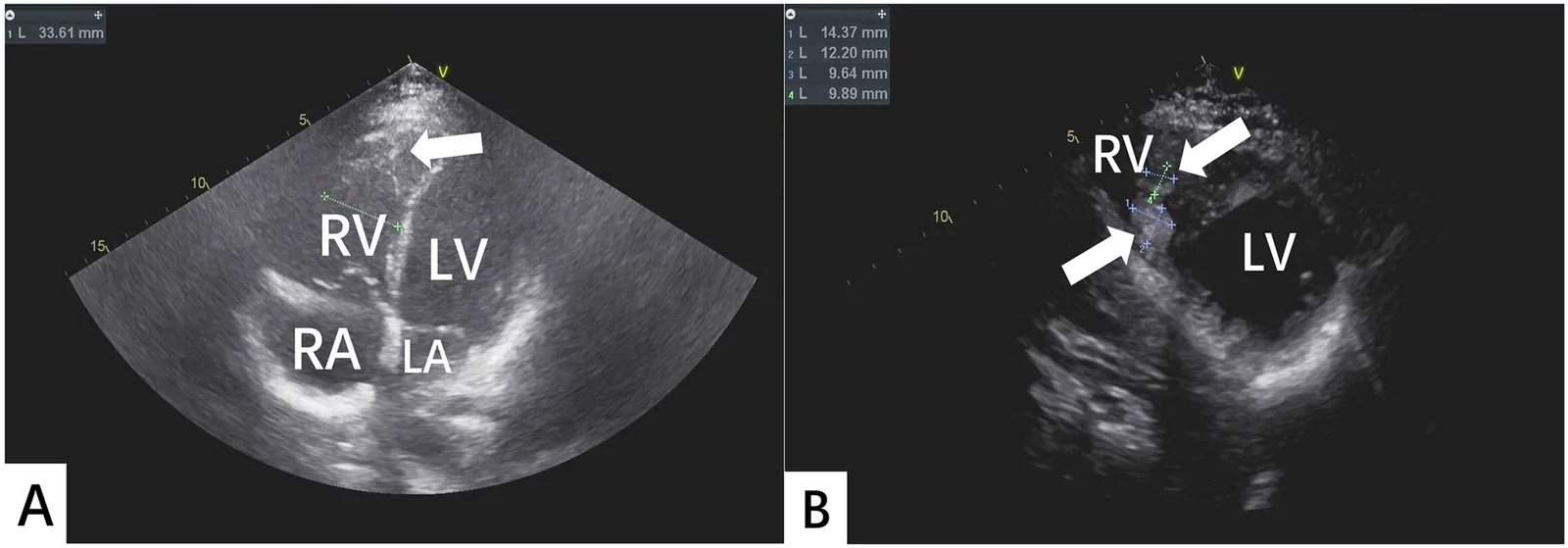
RV, right ventricle; RA, right atrium; LV, left ventricle; LA, left atrium; **(A)** apical four-chamber view. Marked dilatation of the right atrium and right ventricle is observed. A mixed cord-like echo is visualized at the right-ventricular apex accompanied by diffuse myocardial trabeculation (white arrow). **(B)** Transthoracic echocardiography, left-ventricular papillary-muscle-level short-axis view. Two focal hyper-echogenic lesions are attached to the right-ventricular apex (white arrows), suggestive of adherent thrombus.

CMR was performed at our hospital on September 12, 2025. The right ventricular end-diastolic volume indexed to body surface area (RVEDV/BSA) was 121.94 mL/m^2^, the left ventricular end-diastolic volume indexed to body surface area (LVEDV/BSA) was 86.71 mL/m^2^, and the right ventricular ejection fraction (RVEF) was 14%. The scan showed that the right ventricular end-diastolic transverse diameter was approximately 61.4 mm, with thinning and reduced motion of the apical right ventricular wall. CMR-cine sequence demonstrated focal systolic wrinkling of the right ventricular free wall with accordion-like configuration, which may suggest regional systolic dyssynchrony. A low-signal lesion was noted at the right ventricular apex on cine imaging. Late gadolinium enhancement (LGE) imaging showed no delayed enhancement in the right ventricular wall, and the mass at the right ventricular apex was generally unenhanced with only a small area of internal enhancement, potentially indicative of porous thrombus tissue (Figure 3). LGE revealed diffuse epicardial enhancement involving the anterior and anterolateral walls of the left ventricle. These multimodal CMR findings were supportive of ACM complicated by thrombus formation (Figures 4A,B). In terms of imaging differentiation, trabecular hyperplasia is a component of normal or ectopic myocardial structure, whose signal intensity is generally similar to that of normal myocardium and usually presents myocardial-like enhancement on LGE sequence. Most cardiac tumors tend to show early perfusion or enhancement, and malignant lesions are more likely to have prominent enhancement, which helps distinguish them from the right ventricular apical lesion, thrombus and trabecular hyperplasia in this case. In addition, significant discrepancies in right ventricular diameter measurements between echocardiography and CMR are primarily attributed to inherent technical differences in imaging principles, acquisition planes, and scanning protocols between the two modalities, and the semi-quantitative right ventricular diameter derived from 2D echocardiography is not suitable for direct one-to-one comparison with quantitative CMR measurements. We found that the patient had biventricular myocardial involvement with predominant right ventricular impairment. Integrating the patient's clinical manifestations, electrocardiographic features, imaging findings and laboratory examinations, a definitive diagnosis of right ventricular-predominant ACM was established, along with multiple mural thrombi at the right ventricular apex, chronic heart failure, mild anemia and hyperuricemia.

**Figure 3:**
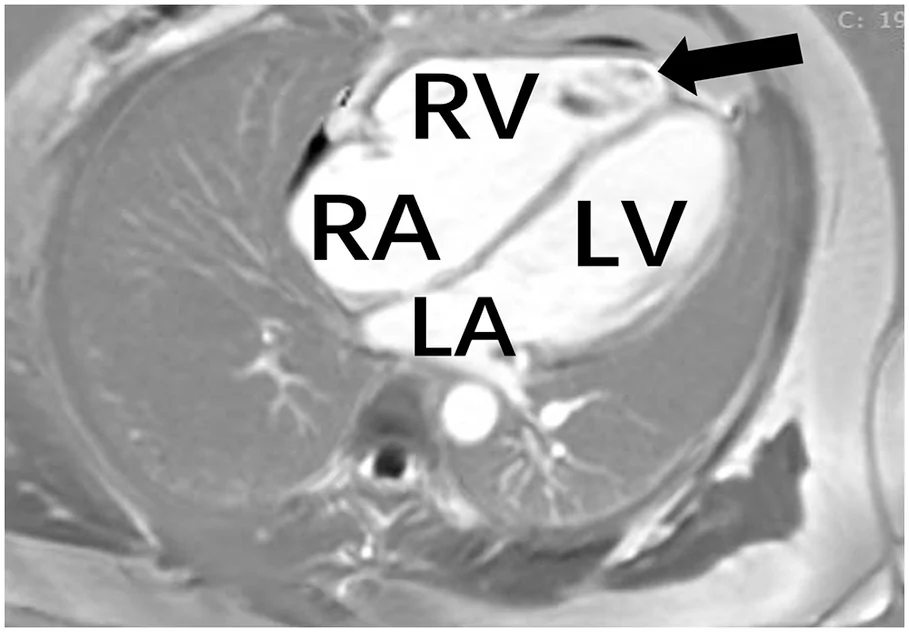
CMR, cardiac magnetic resonance; RV, right ventricle; RA, right atrium; LV, left ventricle; LA, left atrium; late gadolinium-enhanced (LGE) four-chamber CMR view. Black arrow indicates the mass located at the right ventricular apex. There is no delayed gadolinium enhancement within the right-ventricular free wall; the apical mass is largely unenhanced with only focal internal enhancement, which may suggest a loose structure. Diffuse epicardial enhancement can be observed in the anterior and anterolateral walls of the left ventricle, consistent with the imaging features of arrhythmogenic cardiomyopathy (ACM) with suspected thrombus formation.

**Figure 4:**
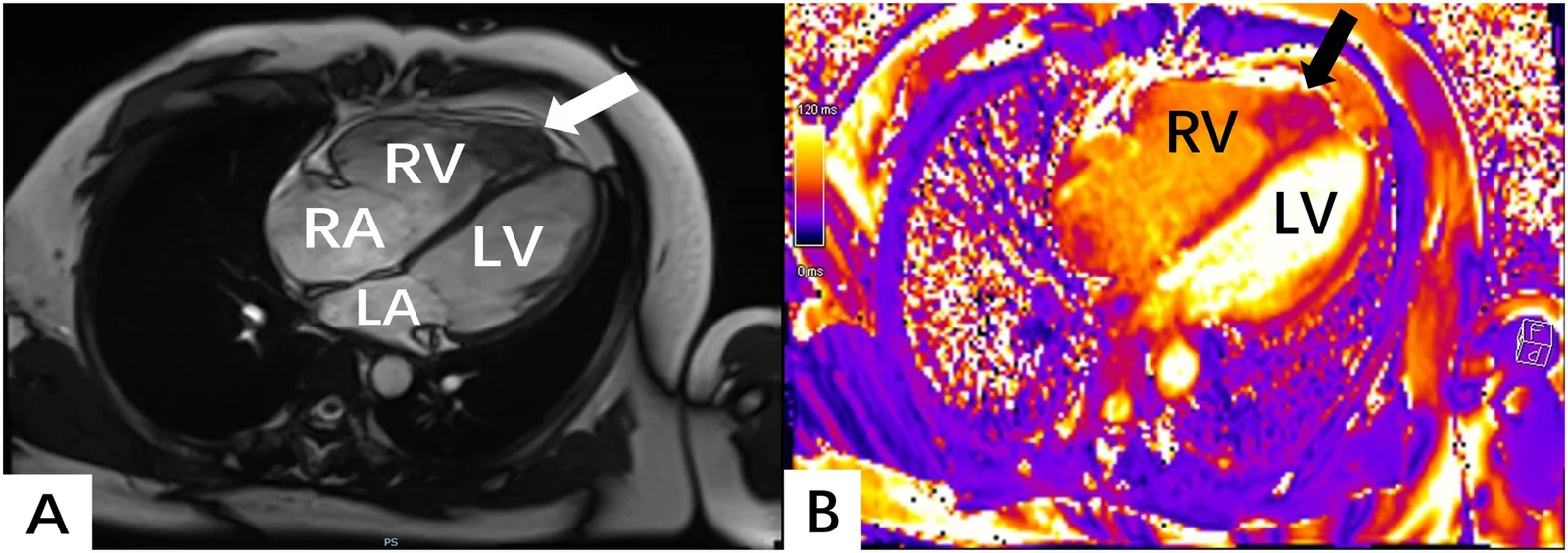
CMR, cardiac magnetic resonance; bSSFP, balanced steady-state free precession; RV, right ventricle; RA, right atrium; LV, left ventricle; LA, left atrium; **(A)** balanced steady-state free-precession (bSSFP) cine sequence (transverse plane, end-diastole). The end-diastolic transverse diameter of the right ventricle is significantly increased. The right ventricular apex shows focal wall thinning with a hypointense lesion (white arrow), consistent with thrombus. **(B)** Pseudo color strain-mapping image obtained by post-processing of CMR-cine sequence. The black arrow indicates the right ventricular apex with altered regional strain.

After admission, the patient received standardized comprehensive symptomatic and etiological treatment. Rivaroxaban 20 mg once daily was administered for in-hospital short-term anticoagulation. The patient maintained normal renal function throughout hospitalization, and no bleeding-related adverse events, such as melena, hematochezia, skin ecchymosis, or petechiae, were observed. Given the patient's normal renal function and low bleeding risk, direct oral anticoagulant monotherapy with rivaroxaban was adopted for initial anticoagulation, and oral anticoagulation was continued after discharge to prevent thrombus progression and secondary embolic events. In addition, spironolactone was prescribed to reduce cardiac load; sacubitril-valsartan and sustained-release metoprolol succinate were used to improve cardiac function and delay adverse myocardial remodeling; folic acid and iron supplements were given to correct anemia for supportive symptomatic management; and benzbromarone was administered for uric acid reduction. After systematic treatment, the patient's clinical symptoms of chest tightness and shortness of breath were markedly relieved. The patient achieved unassisted supine sleeping at night, with significantly improved exercise tolerance compared with admission status (Table 1).

**Table 1:** Timeline of clinical events, diagnostic workup, and treatment.

Timepoint	Event
3 months before admission	Onset of recurrent chest tightness and dyspnea
2 weeks before admission	Progressive worsening of chest tightness and dyspnea
Day 1 (admission)	Physical examination, laboratory tests, chest CT: exclude pulmonary embolism
Day 2	TTE: right ventricular dilatation and apical mural thrombus detected
Day 4	Cardiac MRI: confirmed ACM and intracardiac thrombus
Day 1 onwards	Initiation of rivaroxaban anticoagulation and cardiac supportive therapy
Follow-up imaging	Thrombus resolution, improvement of clinical symptoms

## Discussion

ACM is an inherited cardiomyopathy characterized pathologically by fibrofatty replacement of the myocardium. It demonstrates substantial clinical phenotypic heterogeneity, and concomitant thrombus formation is relatively rare, with an incidence of less than 5% (1). The diagnosis of ACM requires evidence from multiple aspects such as clinical symptoms, electrocardiogram, imaging, and family history. The current diagnosis is mainly based on the 2020 Padua Criteria and the recommendations of the 2023 ESC Guidelines for the Management of Cardiomyopathies (3, 4).

The patient in this case presented with recurrent chest tightness and dyspnea exacerbated by exertion, accompanied by nocturnal dyspnea, which is consistent with the clinical manifestations of cardiac dysfunction in ACM. The early ultrasound diagnosis indicated significant enlargement of the right atrium and right ventricle, diffuse myocardial trabeculation in the right ventricular apex, and multiple hyperechoic masses. CMR further confirmed right ventricular dilatation, apical hypokinesis, and low-signal thrombus. Diffuse epicardial late gadolinium enhancement was observed in the anterior and anterolateral walls of the left ventricle, consistent with the core features of regional myocardial motion and morphological abnormalities (2). The patient had biventricular myocardial involvement with predominant right ventricular impairment.

We believe that the combined application of transthoracic echocardiography and cardiac magnetic resonance is crucial for the diagnosis of ACM. Echocardiography, as the preferred non-invasive imaging method, can display the characteristic structural change of diffuse myocardial trabeculation at the right ventricular apex. At the same time, it can identify the size and attachment site of the thrombus, providing early direct morphological evidence for clinical practice. Studies have shown that echocardiographic parameters have significant prognostic value for patients with ACM. The change in right ventricular FAC < 23% is the strongest predictor of Major Adverse Cardiovascular Events (MACE), and TAPSE < 17 mm and right ventricular dilation are also associated with an increased risk of MACE (5). CMR typically establishes the diagnosis of ACM by demonstrating ventricular dilatation, segmental wall motion abnormalities, and characteristic LGE, which provides critical imaging evidence for ACM diagnosis (6). ACM and Brugada syndrome (BrS) may be confused owing to similar involvement of the right precordial leads, yet they differ fundamentally in structural substrate, electrophysiological features and genetic background. BrS is primarily an ion-channelopathy with generally normal cardiac structure, dynamic electrocardiographic changes, and predisposition to polymorphic ventricular tachycardia or ventricular fibrillation. By contrast, ACM is mainly associated with desmosomal gene mutations, characterized by right-ventricular fibrofatty replacement and structural remodeling. Its ECG manifestations include relatively fixed widespread T-wave inversion, epsilon waves and terminal conduction delay, frequently accompanied by monomorphic ventricular tachycardia. Differential diagnosis requires CMR to assess right-ventricular structural abnormalities as well as genetic testing to identify pathogenic variants (7).

Intracardiac thrombosis is a known complication of ACM, and previous cohort-based studies have identified the right ventricular apex as a frequent site for thrombus formation. Most of the available relevant publications are individual case reports (8). While single thrombus in the right ventricular apex is relatively well-described in the literature, the presence of multiple thrombi localized exclusively to the right ventricular apex, as seen in our patient, is comparatively uncommon. The potential mechanism underlying thrombus formation in this case may be speculated based on Virchow's triad, including blood stasis, endothelial injury, and hypercoagulable state (9). The possible pathophysiological process of thrombosis in the present patient is summarized as follows: replacement of the right ventricular apical myocardium by fibrofatty tissue may lead to local myocardial wall thinning and regional hypokinesia, which further promotes thrombotic events. The formation of diffuse myocardial trabeculation led to abnormal blood flow pathways, which further aggravated the local reduction in blood flow velocity and blood stasis, creating conditions for platelet aggregation and fibrin deposition. The fibrofatty lesions of ACM damage the integrity of the endocardium, exposing subcutaneous collagen fibers and activating the endogenous coagulation pathway. At the same time, abnormal hypertrophy of the myocardial trabeculae at the right ventricular apex may cause local friction damage to the endocardium, thereby exacerbating endothelial dysfunction. The patient has severe left ventricular heart dysfunction and also has mild anemia, both of which can lead to hemodynamic disorders and indirectly promote thrombosis (10).

We followed up the patient regularly for nearly one year after discharge. The patient adhered well to continuous oral rivaroxaban anticoagulation for half a year after discharge without any bleeding-related adverse events. Repeated transthoracic echocardiography performed at an external hospital in April 2026 revealed complete resolution of the intracardiac thrombus, and the patient has continued standardized rivaroxaban treatment thereafter. During the follow-up period, the patient remained clinically stable without malignant arrhythmia, syncope, chest pain, or aggravated heart failure. Only occasional mild chest tightness and dyspnea were noted, which could be spontaneously relieved after rest or oxygen inhalation. Although no recurrent thrombus or acute thromboembolic events occurred during short-term follow-up, the long-term prognosis still requires further continuous observation. In this case, no genetic testing was performed during the patient's hospitalization at our center. Subsequent ACM first-line pathogenic gene panel testing covering six well-established genes, PKP2, DSP, DSG2, DSC2, JUP and TMEM43, was completed at an external hospital, whose report returned normal findings. Moreover, no myocardial pathological examination was performed in this patient, which makes it difficult to completely exclude phenotypically similar diseases such as right ventricular myocardial disarray (6). In addition, although 24-hour Holter monitoring and exercise testing were obtained for arrhythmia risk evaluation, we lacked quantitative PVC burden and morphological features of premature ventricular complexes. These aspects represent the major limitations of the present case report. In future clinical practice, relevant examinations should be completed for patients to confirm the diagnosis and guide screening. A long-term follow-up system should be established to facilitate early detection of thrombus and evaluation of the efficacy of anticoagulant therapy.

## Conclusions

ACM combined with multiple thrombus formation in the right ventricle is comparatively uncommon in clinical practice. Its pathogenesis may be presumably associated with local blood stasis in the right ventricle, endocardial injury, and severe cardiac dysfunction. Echocardiography can accurately identify right ventricular enlargement, abnormal myocardial trabeculation, and thrombus characteristics (11). As highlighted in recent evidence among patients with nondilated left-ventricular cardiomyopathy undergoing ventricular tachycardia ablation, beyond structural and functional evaluation, CMR tissue-characterization imaging can detect myocardial scar burden and identify concealed arrhythmogenic substrates. Although derived from a non-ACM cohort, these imaging features may provide prognostic information for arrhythmia recurrence and support risk stratification and individualized clinical decision-making in AC (12). Clinicians should raise awareness of rare complications of ACM and perform biventricular assessment simultaneously. For such patients, transthoracic echocardiography can be arranged early to screen for intracardiac thrombus, enabling timely intervention and improved long-term prognosis.

## Patient perspective

The patient stated that the multimodal cardiac diagnostic work-up reassured her that the cause of her persistent chest tightness and dyspnea had been fully explored. She recalled worsening exertional chest tightness and nocturnal dyspnea in the weeks before admission and felt relieved upon receiving a definitive diagnosis of arrhythmogenic cardiomyopathy with right-ventricular apical thrombi. She tolerated the combined regimen of anticoagulation and cardiac supportive therapy with minor side-effects and experienced noticeable symptom improvement. She gave written informed consent for case publication, hoping to help clinicians recognize similar presentations early and reduce diagnostic delays for other patients.

## Data Availability

The original contributions presented in the study are included in the article/Supplementary Material, further inquiries can be directed to the corresponding author.
